# Variety Identification of Corn Seeds Based on Hyperspectral Imaging and Residual Mamba 1D CNN

**DOI:** 10.3390/foods14203558

**Published:** 2025-10-18

**Authors:** Guoqing Qi, Chengzhong Liu, Junying Han, Yuqian Zhou, Yongsheng Li, Yalong Wang

**Affiliations:** 1 College of Information Sciences and Technology, Gansu Agricultural University, Lanzhou 730070, China; 2 Crop Research Institute of Gansu Academy of Agricultural Sciences, Lanzhou 730070, China

**Keywords:** seed classification, non-destructive testing, spectral analysis, residual neural networks, crop phenotyping

## Abstract

Maize is a globally important crop, and reliable identification of seed varieties is vital for breeding and quality assurance. To overcome the limitations of conventional methods, this study developed a non-destructive approach integrating hyperspectral imaging (HSI) and deep learning. A Residual Mamba One-Dimensional Convolutional Neural Network (RM1DNet) is proposed, which integrates residual and Mamba modules to enhance feature learning. RM1DNet achieved 94.85% accuracy in classifying 20 maize seed varieties, outperforming traditional classifiers and baseline deep models. These results demonstrate the robustness and efficiency of RM1DNet, highlighting its potential to advance intelligent seed variety identification using hyperspectral data.

## 1. Introduction

Maize (*Zea mays* L.) is one of the world’s major staple crops, widely cultivated for food, feed, and industrial applications. Accurate variety identification is essential for breeding, cultivation optimization, and quality control [1]. However, conventional approaches such as morphological inspection and molecular marker analysis are often time-consuming, labor-intensive, and costly, making them unsuitable for the modern agricultural demand for rapid, non-destructive, and high-throughput identification [2,3,4].

Hyperspectral imaging (HSI) has emerged as a promising alternative, offering the capability to capture both spatial and spectral information across continuous wavelength bands [5,6]. Its rich phenotypic representation makes it particularly suitable for seed variety identification [7]. Recent studies have confirmed the effectiveness of HSI when combined with machine learning and deep learning techniques [8,9,10,11]. For example, Huang et al. applied an LS-SVM model to classify maize seeds, achieving 94.4% accuracy [12], while Fu et al. developed an SSAE-CS-SVM approach with 95.81% accuracy [13]. More recently, Zhang et al. employed a five-layer CNN with mapped hyperspectral features, reaching 96.65% accuracy [14], and Li et al. introduced the ERNet model, which achieved 98.36% accuracy [6]. These findings highlight the rapid advancement of HSI-based methods in crop variety recognition.

Despite these advances, most existing approaches remain dependent on spectral band selection followed by classifier construction. Although techniques such as Successive Projection Algorithm (SPA) and Competitive Adaptive Reweighted Sampling (CARS) improve computational efficiency, they often discard valuable spectral information, resulting in incomplete feature representation. Furthermore, traditional classifiers and even many deep models struggle to capture the complex nonlinear relationships and long-range dependencies inherent in hyperspectral data, thereby limiting their robustness and generalization ability.

To address these limitations, we propose Residual Mamba One-Dimensional Convolutional Neural Network (RM1DNet), an improved framework that integrates residual modules for deep local spectral feature extraction with Mamba modules for efficient global sequence modeling. The main contributions of this study are as follows: (1) designing a novel 1D CNN architecture that combines residual and Mamba mechanisms; (2) systematically comparing RM1DNet with traditional classifiers and baseline deep models on 20 maize varieties; and (3) evaluating the influence of SPA and CARS feature selection on recognition accuracy. In addition, future work will focus on validating the model under different sampling and environmental conditions to further assess its robustness and practical applicability. The results demonstrate that RM1DNet effectively leverages both local and global spectral information, achieving superior accuracy and robustness for maize variety identification based on hyperspectral imaging.

## 2. Materials and Methods

### 2.1. Experimental Materials

All maize seed samples used in this study were provided by the Gansu Academy of Agricultural Sciences. Twenty widely cultivated maize varieties from Northwest China, including R1831, Longsun 632, Longsun 655 (backcross), Sianyu 1483, Ripu 909, and DF899, were selected and labeled sequentially from 0 to 19. All samples were sealed in kraft paper bags and stored in a cool, dry environment to minimize moisture absorption and prevent deterioration. For each variety, 150 intact and undamaged seeds were selected, resulting in a total of 3000 samples for experimentation. The detailed list of maize varieties is provided in Table 1.

### 2.2. Hyperspectral Image Acquisition

#### 2.2.1. Hyperspectral Imaging Systems

**Figure 1:**
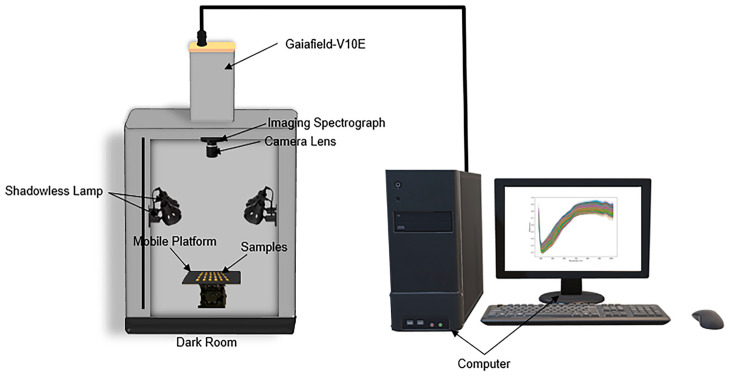
Hyperspectral image acquisition system.

A GaiaField-V10E portable hyperspectral imaging system (Sichuan Dualix Spectral Imaging Technology Co., Ltd., Chengdu, China) was used in this study (Figure 1). The camera covered a spectral range of 380–1018 nm with 320 bands at a spectral resolution of 2.8 nm. The imaging system was equipped with a standardized light source and a dark box to ensure stable acquisition. The push-broom scanning mode was adopted to generate hyperspectral cubes, while system control and calibration were performed using SpecView-50 software. Detailed specifications are provided in the Supplementary Material.

#### 2.2.2. Image Acquisition and Calibration

Prior to image acquisition, the hyperspectral system was preheated for 30 min to minimize baseline drift and then calibrated using SpecView software. Images were captured using the GaiaField-V10E camera under fixed parameters (exposure time: 49 ms; gain: 2; frame rate: 18 Hz; scanning speed: 0.0064 cm/s). For each of the 20 maize varieties, five independent acquisitions were performed, with 30 randomly selected seeds (embryo side up) scanned per acquisition, resulting in 150 seeds per variety and a total of 3000 samples (100 images) [15]. After image acquisition, the raw hyperspectral images were corrected using a black-and-white reference to eliminate dark current noise introduced by the camera. The black-and-white correction equation is shown in Equation (1):

(1) $$I_{c}=\frac{I_{\mathrm{raw}}-I_{\mathrm{dark}}}{I_{\mathrm{white}}-I_{\mathrm{dark}}}$$

Here Iraw represents the raw image, Iwhite represents the white reference image, Idark represents the all-black calibrated image, and Ic represents the corrected image.

### 2.3. Spectral Extraction and Preprocessing

**Figure 2:**
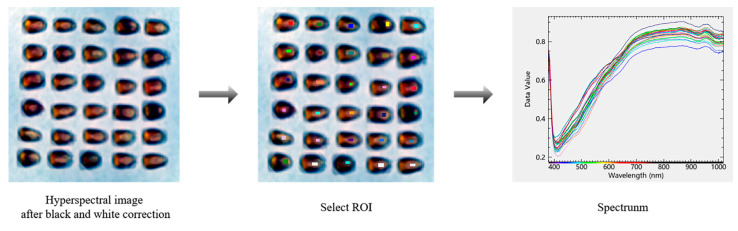
Spectral extraction process.

After black-and-white correction of the original hyperspectral images, the largest possible rectangular region of interest (ROI) was selected on the germinal side of each maize seed using ENVI 5.6 software to minimize the influence of chemical composition heterogeneity. The spectral extraction process is illustrated in Figure 2. The spectral reflectance values of all pixels within the ROI were averaged to obtain the mean spectrum for each seed sample [16].

During spectral acquisition, external interferences such as background noise can compromise data reliability and adversely affect subsequent spectral analysis and model performance. To improve the accuracy and robustness of the model, preprocessing of the raw spectral data is essential to mitigate the impact of these irrelevant factors. In this study, the Savitzky–Golay (SG) smoothing algorithm was employed to preprocess the spectral data. A three-point window was used, including one point on each side of the central point, to form a symmetric kernel.

After spectral feature extraction, all samples were randomly divided into a training set and a test set with a ratio of 8:2 to ensure a fair evaluation of the model’s generalization ability. The partitioning was conducted in a stratified manner based on maize variety labels to maintain class balance. During training, the training subset was used for model optimization, while the test subset was reserved for independent performance evaluation.

### 2.4. Classification Modeling

In this study, a novel convolutional neural network (CNN) architecture, termed RM1DNet, is proposed for the identification of maize varieties. This model incorporates both a residual module and a Mamba module to enhance classification performance. The detailed structure and functional contributions of each component are described below.

#### 2.4.1. ResidualBlock and Mamba Module

**Figure 3:**
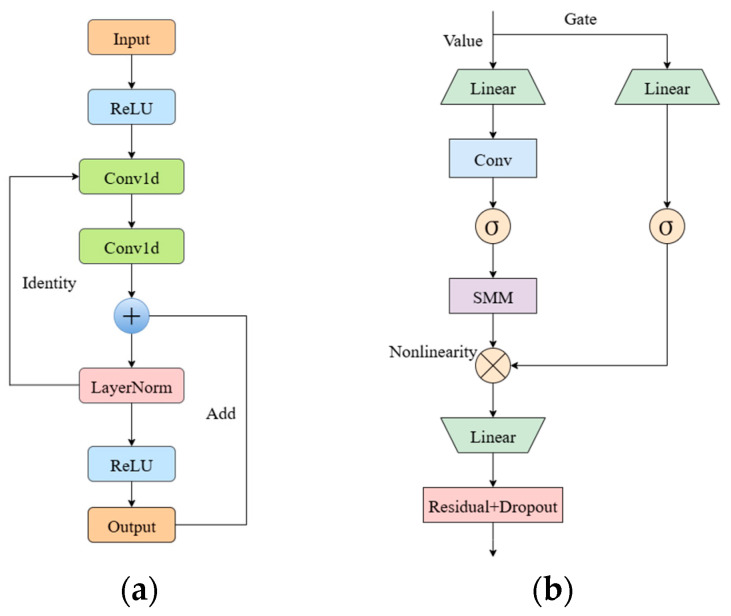
Module structure diagram: (**a**) ResidualBlock, (**b**) Mamba module.

In recent years, convolutional neural networks (CNNs) with residual structures have been widely applied in spectral recognition and image processing, as they enable deeper architectures while alleviating gradient vanishing and network degradation [6,17]. Building on this principle, we designed RM1DNet by integrating three residual modules and a Mamba module to achieve efficient local feature extraction and global dependency modeling (Figure 3).

During convolutional encoding, Residual Blocks are employed to improve feature extraction efficiency and ensure stable gradient flow [18]. Each block consists of two 1D convolution layers, ReLU activation, layer normalization, and a skip connection. The skip connection allows cross-layer feature transfer, while a 1 × 1 convolution is applied if channel dimensions differ. This design accelerates convergence and enhances representational capacity (Figure 3a).

To capture long-range dependencies at a lower computational cost than Transformer-based attention mechanisms, the Mamba module was introduced. Based on a State Space Model (SSM), it incorporates depthwise convolution for local feature extraction, an SSM unit for global modeling, and a gating mechanism to filter informative features selectively. The final output is combined with the input through a residual connection, improving training stability and representational power (Figure 3b).

#### 2.4.2. RM1DNet

**Figure 4:**
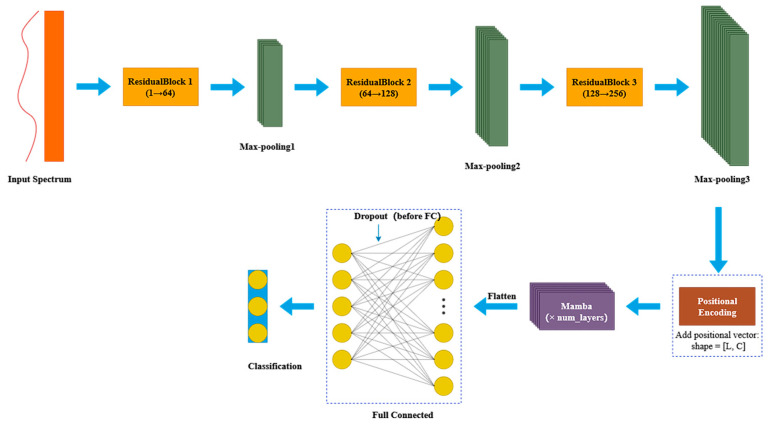
RM1DNet structure diagram.

RM1DNet is a novel hybrid architecture that integrates convolutional feature extraction with state space sequence modeling, aiming to fully exploit both spatial patterns and temporal dependencies within hyperspectral data to enhance the modeling capacity and generalization performance in spectral classification tasks [19]. The model architecture comprises a one-dimensional convolutional neural network (1DCNN), residual modules, Mamba modules, a positional encoder, and a fully connected classification head, as shown in Figure 4.

The front end consists of three Residual Blocks, each containing two 1D convolutional layers and a skip connection, followed by MaxPooling layers for dimensionality reduction. The output channels of the convolutional layers are set to 64, 128, and 256, respectively, facilitating hierarchical feature extraction while mitigating gradient degradation in deep networks. After convolution, the feature maps are reshaped into a [L, B, C] format and augmented with learnable positional encodings before being input to the stacked Mamba modules. Leveraging state space models (SSMs), the Mamba modules efficiently capture long-range dependencies with linear computational complexity, offering improved scalability over conventional Transformer-based designs. The extracted contextual features are subsequently flattened, regularized via dropout, and mapped to class probabilities through a fully connected layer. ReLU activation is applied throughout the network to accelerate convergence and alleviate vanishing gradient issues.

Given the multi-class nature of maize variety classification, this study employs the Label Smoothing (LS) cross-entropy loss function to evaluate model performance. The specific formulation is presented in Equation (2):

(2) $$\mathrm{Loss}=-\sum_{i=1}^{C}\left[\left(1-\varepsilon \right)\cdot y_{i}+\frac{\varepsilon }{C}\right]\cdot \log\left({\hat{y}}_{i}\right)$$

where C is the number of categories; $\varepsilon \in \left[0,1\right]$ is the smoothing factor (usually taken as 0.1); $y_{i}$ is the true label of the i th category; and ${\hat{y}}_{i}$ is the predicted probability of the i th category as output by the model. The smoothed label distribution retains high confidence in the correct class while allocating small uniform weights to the remaining classes, thereby mitigating overfitting and improving generalization.

The model was trained using the Adam optimizer to minimize the loss and maximize classification accuracy. Training was further stabilized and accelerated through a ReduceLROnPlateau scheduler, which reduced the learning rate by half if validation accuracy did not improve over five consecutive epochs, and by applying an early stopping strategy that terminated training if no improvement was observed within ten consecutive epochs.

### 2.5. Baseline Models for Comparison

To rigorously evaluate the performance of RM1DNet, six classical models were selected as baselines, covering both traditional machine learning and deep learning approaches.

Support Vector Machine (SVM): A supervised learning method that constructs optimal hyperplanes in high-dimensional space to maximize inter-class margins, widely used in hyperspectral image classification due to its robustness in small-sample and high-dimensional scenarios.

Extreme Learning Machine (ELM): A feedforward neural network with a single hidden layer, characterized by fast training speed and simple structure, making it suitable for hyperspectral classification [20].

Backpropagation Neural Network (BP): A multi-layer feedforward neural network trained via error backpropagation, providing stronger feature representation than ELM [21].

Long Short-Term Memory (LSTM): A recurrent neural network specialized for modeling sequential data and capturing long-term dependencies in spectral sequences [22].

1D Convolutional Neural Network (1DCNN): Applies convolutional filters along one-dimensional spectral inputs to extract local features, showing strong capability in hyperspectral classification [23].

Residual 1DCNN (Res1DCNN): Enhances 1DCNN with residual connections to mitigate gradient degradation and improve deep spectral feature learning.

Mamba1DCNN: A hybrid variant that combines 1DCNN with Mamba modules to capture both local spectral patterns and global dependencies.

### 2.6. Feature Selection Algorithms

In hyperspectral modeling tasks, feature selection plays a crucial role in reducing dimensionality, eliminating redundancy, and improving the interpretability and stability of classification models. While traditional machine learning methods such as SVM, ELM, and BP generally rely on feature selection to enhance accuracy, its impact on deep learning models remains insufficiently explored [24,25]. To systematically investigate this aspect, two representative algorithms—Successive Projection Algorithm (SPA) and Competitive Adaptive Reweighted Sampling (CARS)—were employed in this study.

**Figure 5:**
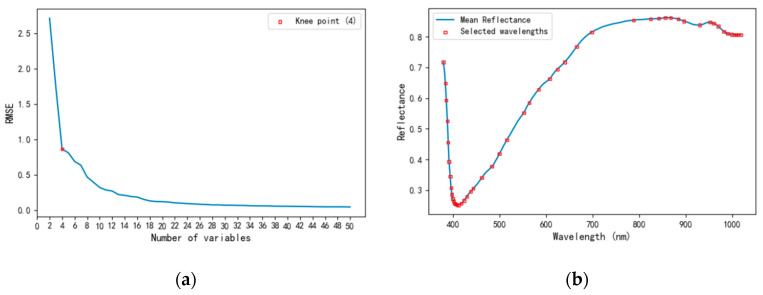
SPA extraction of characteristic wavelengths. (**a**) Number of variables. (**b**) Position of variables.

SPA is a forward-selection-based feature extraction algorithm that incrementally constructs a subset of variables by minimizing linear correlation, thus reducing redundancy [26,27]. The feature selection process using SPA is illustrated in Figure 5. As shown in Figure 5a, the root mean square error (RMSE) of the model decreases sharply with the increasing number of selected variables, with an inflection point observed when four variables are selected. Beyond this point, the reduction in RMSE slows, suggesting diminishing returns. Therefore, a final subset of four wavelengths is chosen to balance model accuracy and simplicity. Figure 5b visualizes the distribution of selected wavelengths along the mean reflectance spectrum.

**Figure 6:**
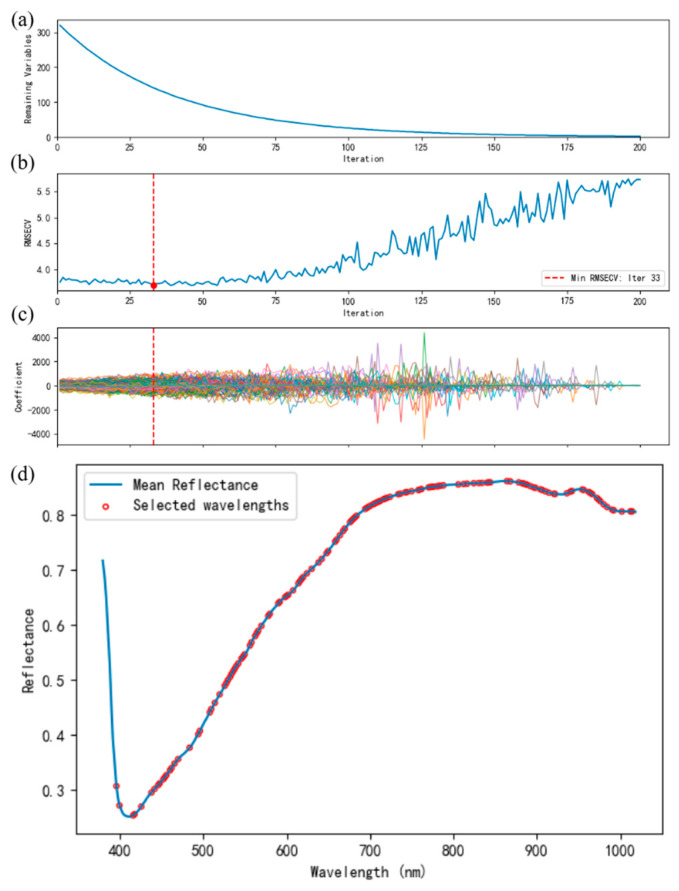
The process of extracting characteristic wavelengths by CARS. (**a**) Preferred number of characteristic wavelength variables. (**b**) Root-mean-square error of cross-validated variances. (**c**) Path diagram of regression coefficients. (**d**) Location of variables.

The CARS algorithm integrates Monte Carlo sampling with partial least squares (PLS) regression to dynamically select spectral bands that most significantly affect model performance [27,28]. The process of CARS feature selection is depicted in Figure 6. As seen in Figure 6a, the number of retained wavelengths drops rapidly as the iteration progresses, representing a shift from coarse to fine selection. Figure 6b displays the cross-validation root mean square error (RMSECV) for each sampling iteration, with the lowest RMSECV occurring at iteration 33, indicating the optimal variable subset. Figure 6c presents the corresponding PLS regression coefficients, while Figure 6d shows the location of the final selected bands on the average spectral reflectance curve, thereby illustrating their spectral response characteristics.

Furthermore, the discriminative wavelengths selected by SPA and CARS correspond to specific absorption features of key biochemical constituents in maize seeds. For instance, SPA selected characteristic wavelengths around 450 nm, 550 nm, 680 nm, and 970 nm. The 450 nm region corresponds to carotenoids and flavonoids associated with seed pigments; the 550–680 nm range is related to protein and chlorophyll absorption; and the near-infrared region around 950–1000 nm reflects C–H and O–H combination bands linked to starch and lipid molecular vibrations. These findings suggest that the selected spectral bands reflect intrinsic biochemical variations—particularly in pigments, proteins, and carbohydrate composition—among different maize varieties. Even without direct biochemical quantification, these associations provide a plausible physiological basis for the model’s discriminative capability [12,14,18,27].

### 2.7. Software Tools

The operating system used in this experiment was Windows 11 64-bit Professional. The CPU was a 13th generation Intel^®^ Core™ i7-13700@2.10 GHz, the GPU was an NVIDIA GeForce RTX 4090 equipped with 24 GB of video memory, and the system memory was 64 GB. Hyperspectral image preprocessing and spectral extraction were performed using ENVI 5.6, where mean spectra from the embryo face were obtained as regions of interest. Model implementation, including RM1DNet, feature selection algorithms (SPA and CARS), traditional classifiers (SVM, ELM, and BP), and the LSTM model, was carried out in PyCharm 2023.3.

## 3. Results

### 3.1. Spectral Characterization

**Figure 7:**
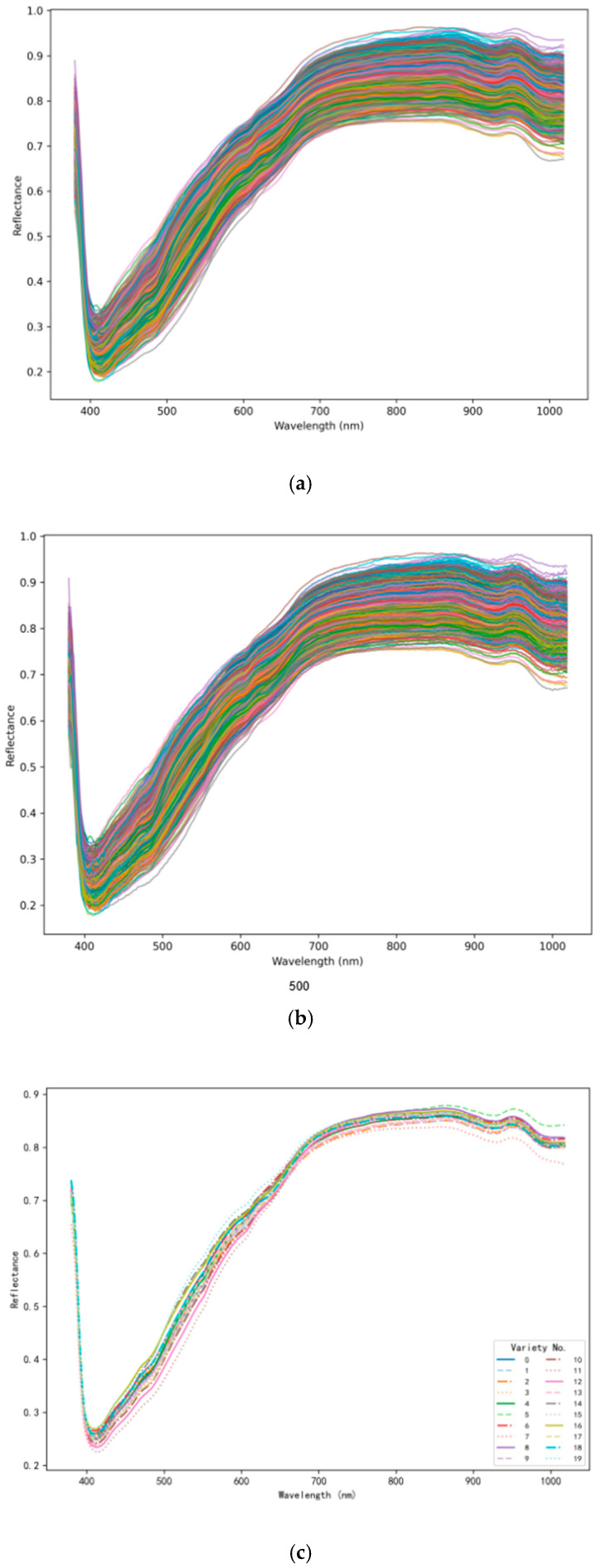
Spectral data: (**a**) all raw spectra, (**b**) all NIR spectra pre-processed by SG, (**c**) average spectra of 20 maize varieties.

Figure 7a presents the raw spectral curves of all samples from the 20 maize varieties. Figure 7b displays the spectral curves after SG smoothing, which effectively eliminated most of the noise in the original spectral data. Figure 7c illustrates the average reflectance spectra of the 20 maize varieties across the 380–1018 nm wavelength range. It can be observed that the overall shape of the spectral curves was largely consistent among all varieties, showing a general trend of increasing reflectance from the visible to the near-infrared (NIR) region. This spectral pattern reflects the characteristic absorption and reflection behavior of maize seeds at different wavelengths. In the 380–550 nm range, reflectance values are relatively low for all varieties, indicating higher light absorption in this region. Between 550 and 750 nm, the reflectance increases rapidly, then stabilizes within the 750–900 nm range, forming a distinct plateau. This plateau region exhibited slight inter-varietal differences, with some varieties showing higher reflectance values while others were slightly lower. Moreover, noticeable variations in reflectance distributions were observed in the 600–750 nm and 900–1018 nm wavelength intervals. These differences suggest that although the overall spectral profiles were similar, specific spectral bands contained discriminative information that differentiated maize varieties.

### 3.2. Exploratory Analysis of Multi-Species Clustering

Before establishing the classification model, an exploratory clustering analysis was conducted on the hyperspectral data to evaluate the intrinsic separability among maize varieties. The original high-dimensional spectral signals were projected into a three-dimensional space using two dimensionality-reduction techniques—Principal Component Analysis (PCA) and Linear Discriminant Analysis (LDA)—to enhance the effectiveness of cluster visualization.

Following PCA, the first three principal components accounted for approximately 40.41% of the total variance, effectively preserving the major variance trends in the original spectral data. In contrast, LDA, as a supervised dimensionality-reduction method, was designed to maximize inter-class variance while minimizing intra-class variance by incorporating category labels. As a result, it provides superior enhancement of spectral separability.

**Figure 8:**
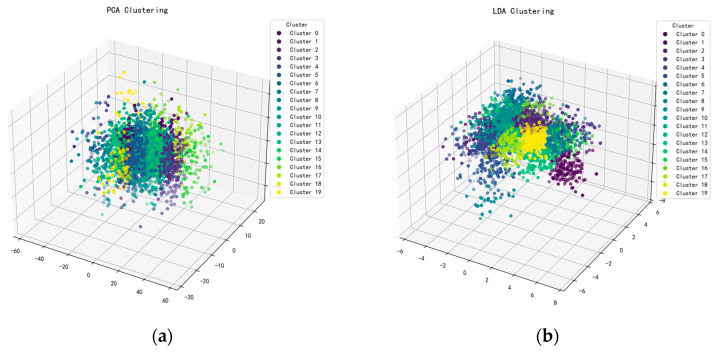
PCA (**a**) and LDA (**b**) 3D clustering diagrams.

Figure 8 illustrates the clustering results after dimensionality reduction. As shown in Figure 8a, the PCA-based 3D scatter plot reveals substantial overlap among data points, with blurred cluster boundaries and ambiguous separations. This indicates that the original spectral feature space contains overlapping distributions across some maize varieties, making it difficult to distinguish them using unsupervised approaches. Conversely, Figure 8b presents the LDA-based clustering visualization, which exhibits clearly separated and well-defined clusters under the guidance of class labels. The enhanced class aggregation and discrimination confirmed the existence of meaningful spectral distinctions among maize varieties.

These exploratory results provided important theoretical support for the subsequent model development. On one hand, PCA serves as a valuable tool for data preprocessing and visual inspection. On the other hand, the pronounced class separability achieved by LDA underscores the feasibility and necessity of incorporating supervised learning strategies—such as deep learning models or ensemble classifiers—during the modeling process. Therefore, this study proposed a novel hybrid architecture integrating RM1DNet. The model’s performance is systematically compared against several representative classification algorithms (e.g., SVM, LSTM) to comprehensively validate its effectiveness and robustness in hyperspectral maize variety identification.

### 3.3. Full-Spectrum Based Classification Model Results

This study systematically evaluated the effectiveness of full-spectrum hyperspectral data in maize variety recognition by comparing traditional machine learning methods, recurrent and convolutional neural networks, and the newly developed RM1DNet model. The tested models included SVM, ELM, BP, LSTM, 1DCNN, Res1DCNN, Mamba1DCNN, and RM1DNet, with results summarized in Table 2.

Among traditional machine learning approaches, SVM demonstrated moderate performance (accuracy 70.57%), while ELM and BP showed limited capability in capturing complex spectral features (accuracies 59.12% and 57.21%, respectively). The LSTM model achieved improved recognition accuracy (85.25%) by leveraging sequence modeling; however, its performance was constrained by spectral redundancy and inter-band correlations.

CNN-based methods exhibited more robust classification. The 1DCNN and Res1DCNN achieved accuracies above 94%, highlighting the benefits of convolutional feature extraction and residual connections for mitigating gradient degradation. Incorporating the Mamba module further enhanced sequence modeling, as demonstrated by Mamba1DCNN (accuracy 94.57%).

The proposed RM1DNet, integrating residual and Mamba modules, achieved the highest performance across all metrics, with an accuracy of 94.85%. This indicates that combining local convolutional feature extraction with global sequence modeling effectively captures subtle spectral variations among maize varieties, improving both accuracy and robustness.

**Figure 9:**
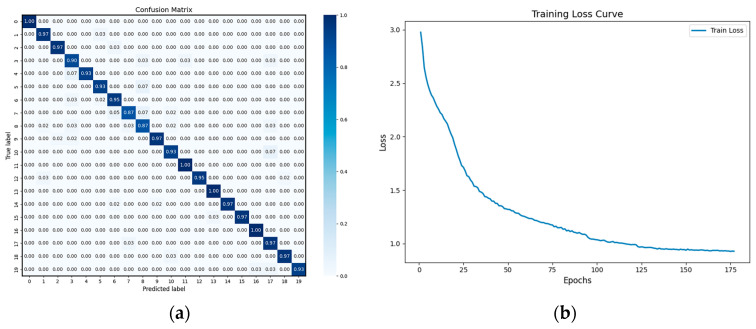
Confusion matrix and loss function. The confusion matrix and loss function of RM1DNet are shown separately. Figure (**a**) shows the confusion matrix and Figure (**b**) shows the loss function, which together demonstrate the classification performance of the model.

Figure 9 presents the confusion matrix and training curve of RM1DNet. The normalized confusion matrix (Figure 9a) indicates that most maize varieties were correctly classified, with classification accuracies exceeding 95%. Minor misclassifications mainly occurred between spectrally similar varieties, such as Longdan656 and Longdan657, which share comparable biochemical compositions. The learning curve (Figure 9b) illustrates a smooth decline in loss and stable convergence, confirming that the model training process was effective and showed no signs of overfitting.

Table 3 details the per-class performance metrics, including precision, recall, F1-score, true positives (TP), false positives (FP), and false negatives (FN). Most classes achieved precision and recall values above 95%, demonstrating that RM1DNet not only achieved high overall accuracy but also performed robustly across all maize varieties.

Overall, these results demonstrate the potential of RM1DNet for precise and robust maize variety classification using hyperspectral data. Nevertheless, future work should consider external validation datasets to further assess generalization, as well as explore the biological basis of spectral differences to better interpret model predictions and guide practical applications.

**Table 3:** Classification performance (Precision, Recall, and F1) for 20 maize varieties using the RM1DNet model.

Corn Kernel Varieties	Precision (%)	Recall (%)	F1 (%)	TP	FP	FN
R1831	100.00	100.00	100.00	150	0	0
Longdan632	98.70	97.30	98.00	146	2	4
Longdan633	98.00	98.70	98.30	148	1	2
Longdan635	97.30	96.00	96.60	144	5	6
Longdan655	98.70	97.30	98.00	146	2	4
Longdan655fanjiao	97.30	98.00	97.60	147	1	3
Xianyu1483	98.70	98.00	98.30	147	3	3
Longdan656	91.50	88.70	90.10	133	14	17
Longdan657	89.30	90.70	90.00	136	12	14
Longdan659	97.30	97.30	97.30	146	2	4
Xianyu335	98.00	98.70	98.30	148	1	2
Longdan636	100.00	100.00	100.00	150	0	0
Longdan2463	96.70	98.00	97.30	147	1	3
Longdan24159	100.00	100.00	100.00	150	0	0
Longdan634	98.00	98.70	98.30	148	2	2
Ruipu909	97.30	96.00	96.60	144	5	6
DF899	100.00	100.00	100.00	150	0	0
Xianyu698	97.30	98.00	97.60	147	1	3
Xianyu1620	96.70	97.30	97.00	146	2	4
Xianyu1516	97.30	98.00	97.60	147	1	3

### 3.4. Classification Model Results Based on Feature Selection

Full-spectrum hyperspectral data often contain substantial redundancy, which can limit the performance of traditional machine learning models that rely heavily on input feature quality. Feature wavelength selection can improve classification accuracy and reduce computational complexity [27]. Compared with conventional algorithms such as SVM, ELM, and BP, 1DCNN exhibits superior automatic feature-extraction capability, thereby reducing the need for manual feature engineering [23].

To evaluate the impact of feature selection, two representative methods—CARS and SPA—were applied to extract informative spectral bands from the original hyperspectral data, yielding 141 and 50 wavelengths, respectively. The classification results based on these selected features are summarized in Table 4. Most models showed decreased or comparable accuracy after feature selection, except for the SPA-LSTM model, which displayed modest improvement. Notably, RM1DNet maintained high performance, achieving test accuracies of 94.14% and 93.27% with CARS- and SPA-selected features, respectively—slightly lower than the 94.85% obtained using the full spectrum.

These results suggest that variations in model performance are influenced by both the intrinsic feature extraction capabilities of each model and the representativeness of the selected wavelengths. RM1DNet consistently demonstrated robust classification accuracy even without feature selection, thanks to its integration of residual convolutional modules with the Mamba state-space mechanism. This architecture enables effective learning of discriminative spectral patterns directly from high-dimensional raw data, reducing the reliance on manual dimensionality reduction.

Overall, RM1DNet provides an efficient and reliable approach for maize variety classification, demonstrating resilience to variations in input feature dimensionality while minimizing the need for explicit feature selection. Future work could further explore the biological basis of spectral differences to enhance model interpretability and practical applicability.

**Table 4:** Classification results of maize varieties based on feature selection methods.

**Models**	**Methods**	**Variables**	**Average Accuracy (%)**	**Highest Accuracy (%)**	**Precision** **(%)**	**Recall** **(%)**	**F1** **(%)**
SVM	CARS	141	70.14	71.58	71.80	71.58	71.60
	SPA	50	63.53	64.75	64.90	64.75	64.78
ELM	CARS	141	59.54	60.50	60.70	60.50	60.55
	SPA	50	59.26	60.17	60.40	60.17	60.20
BP	CARS	141	51.57	52.67	52.80	52.67	52.70
	SPA	50	48.55	49.33	49.50	49.33	49.35
LSTM	CARS	141	84.86	85.83	86.00	85.83	85.85
	SPA	50	88.27	89.17	89.40	89.17	89.20
1DCNN	CARS	141	91.89	92.42	92.63	92.42	92.45
	SPA	50	91.38	91.92	92.02	91.92	91.90
Res1DCNN	CARS	141	93.25	93.67	93.89	93.67	93.67
	SPA	50	92.73	93.17	93.44	93.17	93.16
Mamba 1DCNN	CARS	141	93.64	94.10	94.30	94.10	94.15
	SPA	50	92.93	93.30	93.50	93.30	93.35
RM1DNet	CARS	141	94.14	94.58	94.79	94.58	94.61
	SPA	50	93.27	93.67	93.98	93.67	93.69

## 4. Discussion

This study focused on 20 maize varieties (a total of 3000 seeds), utilizing hyperspectral imaging (380–1018 nm) and developing RM1DNet to investigate the classification performance of different models and the impact of feature-selection strategies on identification accuracy.

In terms of spectral preprocessing, the Savitzky–Golay (SG) smoothing algorithm with a three-point window was employed to mitigate background noise and enhance the reliability of the raw spectral data. Comparative analysis of the preprocessed (Figure 7b) and raw spectra (Figure 7a) showed that SG smoothing effectively preserved the intrinsic spectral characteristics of maize seeds—such as the rapid increase in reflectance between 550 and 750 nm and the stable plateau between 750 and 900 nm—while removing random noise interference. This provided a solid foundation for subsequent model training, consistent with the findings of Chivasa et al. [29], who emphasized that appropriate spectral smoothing enhances the discriminative power of hyperspectral data for crop variety classification.

Regarding model performance, the proposed RM1DNet achieved a test accuracy of 94.85%, significantly outperforming traditional machine learning models (SVM: 70.57%; ELM: 59.12%; BP: 57.21%) and other deep learning architectures (LSTM: 85.25%; 1DCNN: 94.14%; Res1DCNN: 94.39%; Mamba1DCNN: 94.57%). This superior performance arises from RM1DNet’s integrated design: the residual modules mitigate gradient-vanishing issues in deep networks, enabling efficient extraction of local spectral features (e.g., subtle reflectance differences in the 600–750 nm and 900–1018 nm ranges), while the Mamba state-space module captures long-range spectral dependencies with linear computational complexity—thereby overcoming the limitations of conventional CNNs in modeling global sequence information and Transformers in computational efficiency. The confusion matrix (Figure 9a) further confirmed the robustness of RM1DNet, showing 100% classification accuracy for varieties such as R1831 (No. 0) and DF899 (No. 16), although minor misclassifications occurred between spectrally similar varieties (e.g., Longdan656 (No. 7) and Longdan657 (No. 8)), likely due to overlapping biochemical compositions [12].

Compared with existing studies on maize variety classification, this work presents three key advancements. First, whereas Fu et al. [13] achieved 95.81% accuracy for four maize varieties using the SSAE-CS-SVM model, this study successfully classified 20 varieties—reflecting a broader diversity representative of real agricultural production—while maintaining high accuracy. Second, compared with Li et al. [6], who relied on feature engineering for ERNet (98.36% accuracy), RM1DNet requires no manual feature selection, thereby reducing subjective bias and information loss caused by heuristic filtering [17]. Feature-selection experiments (SPA, CARS) showed that traditional models (e.g., SVM) suffered significant accuracy declines when using selected features (SPA: 63.53% vs. full spectrum: 70.57%), whereas RM1DNet retained 93.27–94.14% accuracy, demonstrating stronger adaptability to high-dimensional data and reduced reliance on preprocessing workflows [30].

This study also has certain limitations. First, the hyperspectral data were collected using a single system, and the model’s performance across broader spectral ranges remains to be validated. Second, all samples were collected from Northwest China and stored under uniform conditions; therefore, model generalization to seeds from other regions or under varying storage environments (e.g., high humidity) requires further verification [31]. Only spectral data were used; integrating hyperspectral information with morphological or molecular markers could further reduce the misclassification of spectrally similar varieties [19]. Future studies will address these limitations by extending spectral ranges, diversifying sample sources, and exploring multimodal data fusion.

## 5. Conclusions

This study established a non-destructive framework for maize variety identification based on hyperspectral imaging (380–1018 nm) and deep learning. Using 20 widely cultivated maize varieties (a total of 3000 seeds) from Northwest China, the framework directly processed full-spectrum data (320 bands), thereby minimizing information loss due to dimensionality reduction and providing a more comprehensive evaluation of spectral diversity.

To overcome the limitations of traditional machine learning methods (e.g., SVM, BP) in handling high-dimensional spectral data, a novel model, RM1DNet, was developed by integrating residual modules with the Mamba state-space mechanism. Experimental results demonstrated that RM1DNet achieved a test accuracy of 94.85%, outperforming all seven baseline models. These findings indicate that combining local convolutional feature extraction with global sequence modeling effectively captures subtle spectral differences among maize varieties.

Feature-selection experiments (SPA and CARS) further confirmed the robustness of RM1DNet, which maintained high accuracy (93.27–94.14%) even when trained on reduced feature subsets. This suggests that the proposed architecture is capable of learning discriminative spectral features without relying heavily on manual wavelength selection.

Future research will extend this work by (1) applying hyperspectral systems with broader spectral ranges to assess model adaptability; (2) incorporating maize samples collected under diverse geographical and storage conditions to improve generalization; and (3) exploring multimodal fusion (hyperspectral + RGB imaging) to address challenges in distinguishing spectrally similar varieties.

## Figures and Tables

**Table 1:** Maize varieties.

No.	Name	No.	Name
0	R1831	10	Xianyu335
1	Longdan632	11	Longdan636
2	Longdan633	12	Longdan2463
3	Longdan635	13	Longdan24159
4	Longdan655	14	Longdan634
5	Longdan655fanjiao	15	Ruipu909
6	Xianyu1483	16	DF899
7	Longdan656	17	Xianyu698
8	Longdan657	18	Xianyu1620
9	Longdan659	19	Xianyu1516

**Table 2:** Results of full-spectrum based classification of maize varieties.

Models	Average Accuracy (%)	Highest Accuracy (%)	Precision (%)	Recall (%)	F1 (%)
SVM	70.57	72.21	73.10	72.21	72.15
ELM	59.12	60.50	61.20	60.50	60.40
BP	57.21	58.50	58.80	58.50	58.55
LSTM	85.25	86.42	86.70	86.42	86.45
1DCNN	94.14	94.58	94.82	94.58	94.61
Res1DCNN	94.39	94.83	95.04	94.83	94.84
Mamba1DCNN	94.57	94.95	95.12	94.95	94.96
RM1DNet	94.85	95.25	95.40	95.25	95.25

## Data Availability

The data presented in this study are available on request from the corresponding author due to the data being collected and obtained by individuals.

## Supplementary Materials

The following supporting information can be downloaded at: https://www.mdpi.com/article/10.3390/foods14203558/s1, Table S1: Hyperspectral Imaging System Specifications; Table S2: Image Acquisition Parameters; Figure S3: Example Spectra; Table S4: Model Implementation Details.
