# Supplementary material for: Variety Identification of Corn Seeds Based on Hyperspectral Imaging and Residual Mamba 1D CNN
Source: Foods. 2025 Oct 18;14(20):3558. doi: 10.3390/foods14203558 (PMC12562418; doi:10.3390/foods14203558)
Supplement: Supplementary file 1 [file foods-14-03558-s001.zip › foods-3899040-supplementary.pdf]

S1. Hyperspectral Imaging System Specifications

The GaiaField-V10E portable hyperspectral imaging system (Sichuan Double Alix Spectral Imaging Technology Co., Ltd.) used in this study has the following specifications:

- Spectral range: 380–1018 nm
- Spectral bands: 320
- Spectral resolution: 2.8 nm
- Imaging mode: Push-scan
- Detector: SCMOS, 2048×2048 pixels, 14-bit dynamic range
- Numerical aperture: F/2.4
- Slit size: 30 mm × 14.2 mm
- Light source: Standardized shadowless lamps
- Control software: SpecView
- Calibration targets: HSI-CT-150×150 standard white board, dark current reference

All raw images were corrected using black-and-white calibration to remove dark current noise and normalize reflectance values, following Eq. (1) in the main text.

S2. Image Acquisition Parameters

Parameter	Value
Exposure time	49 ms
Gain coefficient	2
Frame rate	18 Hz
Scanning platform speed	0.0064 cm/s
Preheating time	30 min
Seeds per acquisition	30
Acquisitions per variety	5
Total seeds per variety	150
Total seeds	3,000
Total hyperspectral images	100

Seeds were placed embryo-side up in the dark box. After each acquisition, seeds were returned to the sample bag and shaken to ensure randomization before the next acquisition.

S3. Example Spectra

Representative mean spectra extracted from the region of interest (ROI) on the germinal side of maize seeds are shown in Fig. S1. Spectral preprocessing was performed using the Savitzky-Golay (SG) smoothing algorithm with a window size of 3 points.

Figure S1. Mean spectral reflectance curves for selected maize varieties after SG smoothing.

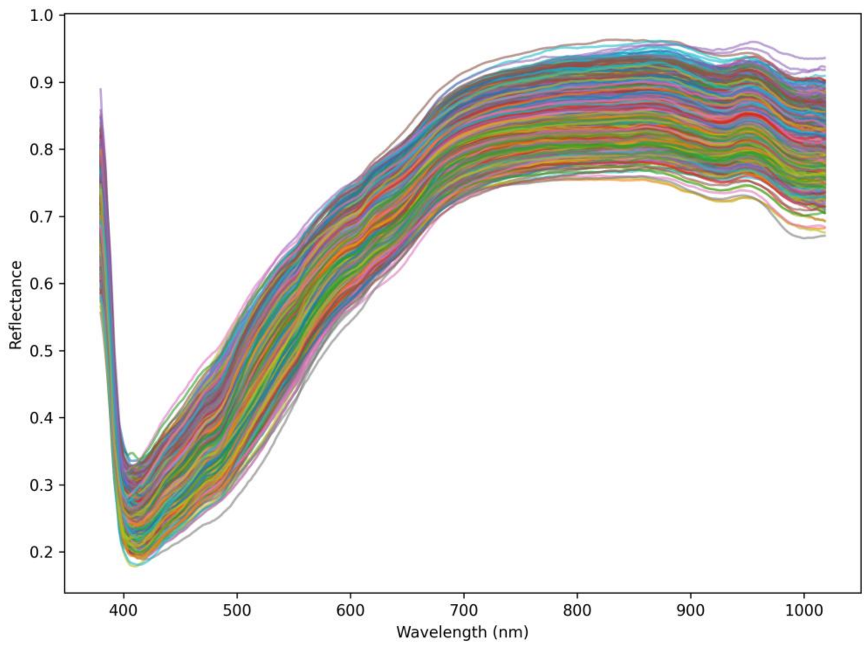

S4. Model Implementation Details

- RM1DNet: Implemented in PyTorch 2.1 using Python 3.11 within PyCharm 2023.3.
- Hardware environment:
  - CPU: Intel® Core™ i7-13700 @ 2.10 GHz
  - GPU: NVIDIA GeForce RTX 4090, 24 GB VRAM
  - RAM: 64 GB
- Training parameters:
  - Optimizer: Adam
  - Initial learning rate: 1e-5
  - Weight decay: 1e-3
  - Batch size: 32
  - Maximum epochs: 300
  - Learning rate scheduler: ReduceLROnPlateau (halving LR if no improvement over 5 epochs)
  - Early stopping: Stop if no improvement in 10 epochs
- Baseline models (SVM, ELM, BP, LSTM, 1DCNN, Res1DCNN, Mamba1DCNN) were trained under identical conditions for fair comparison.
